# The complete chloroplast genome of *Sedum emarginatum* (Crassulaceae)

**DOI:** 10.1080/23802359.2020.1797567

**Published:** 2020-07-29

**Authors:** Hong Chang, Yigong Tang, Xiaoting Xu

**Affiliations:** Key Laboratory of Bio-Resource and Eco-Environment of Ministry of Education, College of Life Sciences, Sichuan University, Chengdu, PR China

**Keywords:** *Sedum emarginatum*, chloroplast genome, phylogenetic analysis

## Abstract

*Sedum emarginatum* Migo is a perennial herb endemic to China. Here, we assembled and characterized the complete chloroplast genome of *S*. *emarginatum* using Illumina sequencing data. Our assembled chloroplast genome is 149,188 base pairs (bp) in length, containing a large single-copy (LSC) region of 81,399 bp, a small single-copy (SSC) region of 16,721 bp, and two inverted repeat (IR) regions of 25,534 bp. In addition, the chloroplast genome possesses a total of 127 genes, including 82 protein-coding genes, eight rRNA genes, and 37 tRNA genes.

*Sedum emarginatum* Migo is a perennial herb of the family Crassulaceae (Mort et al. 2001). This species is native to China, and can be found in many provinces of southern China. In addition, *S*. *emarginatum* is commonly grown as an ornamental and medicinal plant (Bai et al. 2019). Here, we assembled and characterized the complete chloroplast genome of *S. emarginatum* using Illumina sequencing data, providing a valuable genomic resource for future studies.

Fresh leaves of *S*. *emarginatum* were collected from Sichuan Province, China (geographic coordinate: 30°37′55.12″ N, 103°10′42.52″ E), and a voucher specimen (ZYC190510) was deposited in the Sichuan University Herbarium (SZ). The total genomic DNA was extracted using a modified CTAB method (Doyle and Doyle 1987). One library with an insertion size of ∼300 bp was prepared and sequenced using the Illumina platform. The filtered reads were assembled using NOVOPlasty v2.7.2 (Dierckxsens et al. 2017), with the chloroplast genome of *Sedum sarmentosum* Bunge (Dong et al. 2013) as the reference. The assembled chloroplast genome was annotated using Plann v1.1 (Huang and Cronk 2015) and manually inspected using Geneious v11.0.3 (Kearse et al. 2012). The chloroplast genome together with gene annotations was submitted to GenBank (Sayers et al. 2020) under the accession number of MT680404. To further investigate the phylogenetic placement of *S*. *emarginatum*, a maximum likelihood tree was constructed by including nine additional Crassulaceae species and one Penthoraceae species. Here, DNA sequences from these 11 chloroplast genomes were first aligned using MAFFT v1.3.13 (Katoh and Standley 2013), and a phylogenetic tree was then constructed using RAxML v8.2.11 (Stamatakis 2014) with 1000 rapid bootstrap replicates (Stamatakis et al. 2008).

The chloroplast genome of *S*. *emarginatum* is 149,188 base pairs (bp) in legnth, containing a large single-copy (LSC) region of 81,399 bp, a small single-copy (SSC) region of 16,721 bp, and two inverted repeat (IR) regions of 25,534 bp. The overall GC-content of the chloroplast genome is 37.8%, and the GC-contents of the LSC, SSC, and IR regions are 35.7%, 31.8%, and 43.0%, respectively. In addition, the chloroplast genome of *S*. *emarginatum* possesses a total of 127 genes, including 82 protein-coding genes, eight rRNA genes, and 37 tRNA genes. In addition, our phylogenetic analyses show that *S*. *emarginatum* is closely related to *S*. *sarmentosum* with a 100% bootstrap support (Figure 1).

**Figure 1.**
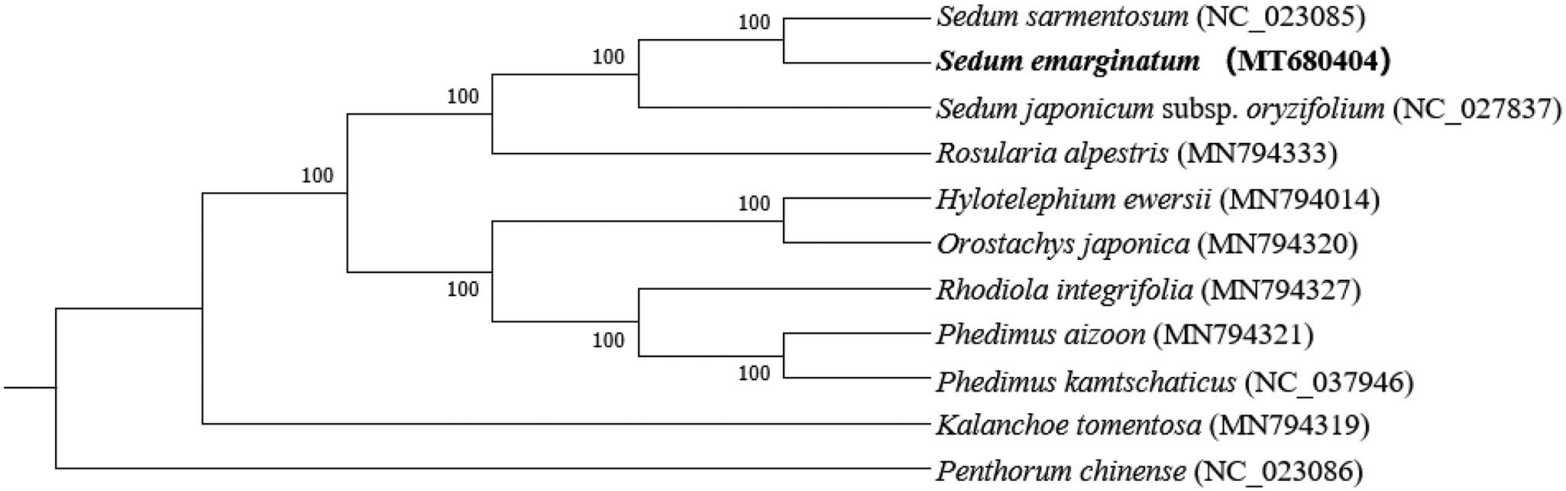
Phylogenetic relationships of *Sedum emarginatum* based on complete chloroplast genome sequences. Bootstrap percentages and GenBank accession numbers are indicated for each branch and taxon, respectively.

## Data Availability

The data that support the finding of this study are open available in NCBI at http://www.ncbi.nlm.nih.gov/, reference number [MT680404], or available from the corresponding author.
